# Dissemination of Research Findings to Research Participants Living with HIV in Rural Uganda: Challenges and Rewards

**DOI:** 10.1371/journal.pmed.1001397

**Published:** 2013-03-05

**Authors:** Anna Baylor, Conrad Muzoora, Mwebsa Bwana, Annet Kembabazi, Jessica E. Haberer, Lynn T. Matthews, Alexander C. Tsai, Peter W. Hunt, Jeffrey N. Martin, David R. Bangsberg

**Affiliations:** 1 Massachusetts General Hospital Center for Global Health, Boston, Massachusetts, United States of America; 2 Mbarara University of Science and Technology, Mbarara, Uganda; 3 Harvard Medical School, Boston, Massachusetts, United States of America; 4 University of California San Francisco, San Francisco, California, United States of America; 5 Ragon Institute of MGH, MIT and Harvard, Boston, Massachusetts, United States of America; 6 Harvard School of Public Health, Department of Global Health and Populations, Boston, Massachusetts, United States of America

## Abstract

David Bangsberg and colleagues explore the challenges and rewards of sharing research findings with participants living with HIV enrolled in observational research in rural sub-Saharan Africa.

**Summary Points**

- Sharing research findings with participants living with HIV enrolled in observational research in rural sub-Saharan Africa presents significant challenges with respect to literacy, language, logistics, and confidentiality.
- Preparation of findings into the local language improved communication between investigators and staff.
- Oral dissemination to 466 participants during a meeting modeled after a traditional wedding event was enthusiastically received by participants, was a rewarding experience for the research team, and identified new areas for investigation.

Community participatory research emphasizes communication of study findings to research participants of vulnerable populations [1]. Most dissemination activities in sub-Saharan Africa have occurred after the completion (or termination) of randomized clinical trials of a defined intervention [2]–[4]. Sharing research findings with participants during observational research can avoid therapeutic misconception [5] as well as evaluate the validity of research involving knowledge, attitudes, or behavior through a “member check” procedure in which investigators conduct interviews regarding the relevancy and saliency of their findings [6]. Nonetheless, the communication of research findings to participants living with HIV enrolled in observational research in a rural sub-Saharan African setting is less straightforward and presents significant challenges with respect to literacy, language, logistics, and confidentiality.

We communicated research findings to 540 participants enrolled in an ongoing 7-year prospective cohort study of HIV treatment in Mbarara, Uganda. We had not organized prior dissemination meetings. This participant dissemination meeting was motivated in part by feedback from a study participant, who said: “You have been asking me questions and taking my blood for years but I do not know anything about what you have found.” Herein, we describe our first approach to the preparation, logistics, confidentiality concerns, format, participant responses, and follow-up of the dissemination process.

Study participants in the Uganda Antiretroviral Rural Treatment Outcomes (UARTO) cohort study are adults living with HIV initiating antiretroviral therapy (ART) at the Immune Suppression Syndrome (ISS) Clinic affiliated with the Mbarara University of Science and Technology (MUST) in rural, southwestern Uganda. The primary objective of the study is to examine the social, behavioral, and economic correlates of long-term adherence to ART using wireless real-time adherence monitoring and to determine the extent to which adherence behavior affects biologic outcomes.

We faced several challenges in reporting research findings to this population. Complicated scientific concepts needed to be distilled into simple core messages that could be easily understood—and not misinterpreted—by all participants in the local language (Runyankole), including those with limited formal education. We believed written dissemination would be ineffective since many participants cannot read, and individual oral communication to more than 500 participants is overly resource intensive. Group communication would be complex both in respect to confidentiality and logistics of convening study participants who live within a 60-km radius catchment area in the context of a poor transportation infrastructure. Regardless of the mode and format of communication, accurate translation of scientific concepts from English into Runyankole can be a complicated and lengthy process due to lack of scientific terminology.

## Step One: Exploring Acceptability, Format, and Content with Participants

Each of seven research assistants informally interviewed five to ten of their participants during routine study visits to ask them if they would attend a meeting of the entire cohort and discussed preferred format, venue, and food. The research assistants explained that only participants, research staff, and officials would be included, that the venue would be private, and that no photos or journalists would be allowed. The research assistants explored concerns about confidentiality, specifically the issue of participants seeing one another at the event. The vast majority of participants were comfortable with these protections and assurances. Most participants were willing to attend despite the inherent loss of confidentiality to other participants. As one participant stated, “Well, they are living with this disease, too, and so what can they think about me?”

The contents of the dissemination conference were determined by research assistants' probing conversations with participants. The participants' concerns ranged from the desire to know more about their lab results, to food supplements, to questions regarding planning for pregnancy.

## Step Two: Ethics Review

The dissemination meeting was discussed in advance with the chairs of the Mbarara University of Science and Technology Institutional Review Committee (MUST IRC); the assistant executive secretary of the Uganda National Council of Science and Technology (UNCST), a national agency for the oversight of all research conducted in Uganda); and the Partners Human Research Committee. Participants and human subjects committee chairpersons were informed that we would take reasonable measures to protect confidentiality and that participation was entirely voluntary, but that we could not guarantee confidentiality. Local human subjects committee and UNCST leadership were invited because the idea for the conference took shape during an informal discussion with the UNCST assistant executive secretary about balancing the need for dissemination and protections against inadvertent HIVserostatus disclosure. We discussed the conference in advance with the chair of the Partners and MUST human subjects committees to confirm that there was no objection to having the event.

## Step Three: Preparation of Findings and Content

Each study co-investigator was partnered with one or two study research assistants to develop the content of their presentation. Each of ten co-investigators, both Ugandan and North American, prepared a 15-minute summary of findings related to their area of expertise. The research assistants then undertook the time-intensive task of translating the material from English to Runyankole. Special attention was devoted to communicating at a level that would be meaningful to the study participants, while still scientifically accurate. In the process, we recognized several ambiguities in translation for common questions. We then undertook the standardized translation of scientific terms into Runyankole and intend to incorporate these into the research protocol and consent forms.

The content included findings published over the prior 7 years as well as preliminary findings currently in preparation for publication. Topics included: 1) why people missed HIV antiretroviral medication doses, with particular attention to how social support helps people overcome structural and economic barriers to adherence [7],[8]; 2) the consequences of short-term interruptions in treatment [9]; 3) how the real-time adherence monitor functions and how specific improvements in the device have led to less frequent communication failures (and, therefore, less frequent home visits to collect blood specimens) [10],[11]; 4) how viral suppression, co-infections, and viral rebound affect the recovery of the immune system, and how this affects survival and living with HIV [12]; 5) how the collection of stool helps us understand interactions between micro-organisms in the intestine, HIV, and the immune system; 6) the negative impacts of alcohol on adherence, and how food insecurity affects many aspects of life, including adherence [13]–[16]; 7) how depression compounds HIV stigma, and how both depression and stigma prevent people from accessing social support to overcome structural barriers to sustained ART adherence [13]; and 8) how sexual behavior and fertility rates change as physical and mental health improve on ART [17],[18].

## Step Four: Invitation

All participants were invited to the event. Each research assistant called or visited approximately 80 participants to invite them. These invitations were followed by written letters signed by the Ugandan and North American principal investigators to formalize the invitations. The research assistants recorded invitation acceptance, concerns about the meeting, and transport requirements to attend the meeting.

Among 540 currently active participants, we were unable to contact 53 of them, the majority of whom are known to be lost to follow-up in the cohort. A total of 477 participants were successfully contacted, and an additional ten heard about the event from other participants. Twelve participants declined the invitation: six declined for fear of disclosure and stigma, and six declined due to inflexible work and personal obligations.

The assistant executive secretary of UNCST, chair of MUST-IRC, dean of the MUST Faculty of Medicine, the MUST vice chancellor, the US National Institute of Mental Health (NIMH) project officer, the ISS clinic director, and several ISS Clinic staff were invited. All attended except for the MUST vice chancellor, who sent the MUST registrar as his representative.

## Step Five: Facilitating Transportation and Arrival

Each participant was offered the choice of transportation to the meeting by a study driver or reimbursement for public transportation. Participants using public transportation gathered at the clinic in the early morning and were shuttled to the venue. The vast majority of participants chose public transportation. They arrived earlier than requested and dressed in traditional, formal Runyankole attire. Research staff individually greeted each participant at a decorated entrance. Of the 475 participants who accepted the invitation, nine did not attend, for a total of 466 study participants in attendance.

## Step Six: Program Agenda and Format

The event was modeled after a typical Runyankole wedding celebration to create a familiar and comfortable setting, which included a raised and covered stage with rows of covered seating arranged perpendicular to the stage on either side. Decorations consisted of streamers and floral arrangements.

The meeting was facilitated by a Ugandan physician well known to the study participants through his work at the clinic. For each of several topics, he introduced the investigator and the research assistant partners, who approached the stage together. The research assistants presented the information in Runyankole with the investigators standing at their sides. Throughout the meeting, research staff translated and recorded questions raised by participants. Questions were collected by the facilitator and communicated to the investigators. The investigators selected representative questions and answered in English, and these answers were then translated orally into Runyankole for the study participants.

As is customary in southwest Uganda, there were two breaks in the presentations for entertainment. UARTO research assistants performed a local Bakiga dance that many participants and investigators joined spontaneously. A song and drumming performance group, comprised of 15 patients from the ISS Clinic, performed in partnership with a North American researcher (PWH) who accompanied them on trumpet. They sang two original songs about youth and HIV prevention as well as the history of the UARTO study.

**Figure 1:**
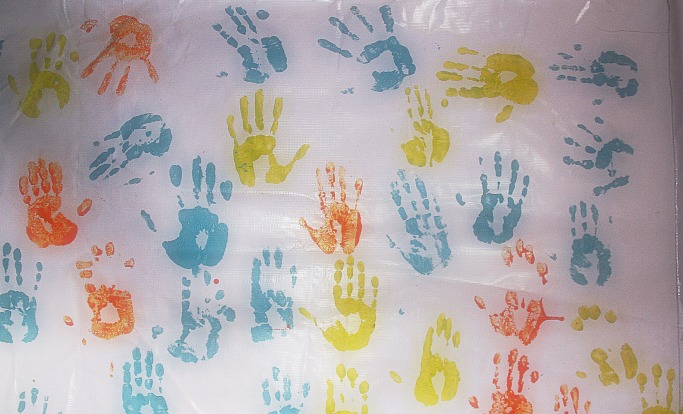
Handprints made during dissemination project.

After the presentation and questions, all participants; the research staff; representatives from MUST IRC, UNCST, and MUST; and the Ugandan and North American investigators shared a traditional Ugandan buffet lunch. Participants, investigators, and guests were invited to leave their handprint in bright colors on one of several large banners to symbolize the gathering (Figure 1). Participants were given certificates of completion with their printed names, and both the Ugandan and North American principal investigators signed each certificate. Permission to print the named certificate was requested of each participant beforehand. All participants chose to have their names printed on the certificate rather than have a certificate without a name. Transportation reimbursements were then provided to each participant. The co-investigators and staff conducted an informal evaluation of the event during the reception. Themes were discussed that evening and the following day.

## Discussion

The informal response from participants was overwhelmingly positive and supported by 98% attendance of those who accepted the invitation. According to research assistants, there is a consensus among participants that the dissemination event should be held annually. Several participants stated that the event made them feel like participants in the research rather than research “subjects.” More importantly, several participants stated that the event made them feel respected, and they personally thanked the North American principal investigator “for honoring us today.”

The handprint banners were hung in the research offices and the phlebotomy room in Mbarara and at the MGH Center for Global Health office in Boston. Framed segments of the banners were given to MUST leadership, the MUST IRC chairperson, UARTO investigators, the NIMH project officer, and the private donors who sponsored the event.

The process of preparing the scientific presentations with the research staff highlighted subtle aspects of English–Runyankole language translation. While our study instruments are routinely translated and then back translated, we identified additional points of potential miscommunication. During this process, we learned that study participants often ask questions that research assistants are unprepared and unauthorized to discuss. The dissemination conference provided a formal mechanism for the research assistants to share participants' concerns and questions with the entire investigator team. As a result of the meeting, we now routinely review questions generated by participants in order to identify additional ambiguities and generate accurate standardized responses. We are also pursuing several areas of investigation based on the participants' feedback, including interventions related to income generation, food security, safely achieving reproductive goals, and adherence support. We plan to invite participants to lead more detailed discussions on their perceptions of high impact research topics during future disseminations. We also plan to invite additional stakeholders, including clinic sponsors, to future conferences. While we do not suggest that dissemination conferences like this should be standard for all studies since we did not include a formal evaluation, we suggest it as a model for future evaluation.

In summary, the dissemination of our scientific findings to a cohort of people with HIV living in rural Uganda was highly rewarding for participants, research staff, and investigators. It improved communication between participants and research staff, strengthened the relationship between research staff and investigators, and created a sense of community among participants. Finally, the event generated a research agenda directly from those most affected by HIV in a rural, resource-constrained setting. We recommend this format as a guide to dissemination of study findings to study participants in similar settings.

**Box 1. Representative Questions from UARTO Participants**

**Economic assistance & food insecurity**

- Is it possible to give us the starting capital so as to do business and buy things like food or cater for our transport?
- What do I do to have food, if am weak and have no social support but at the same time I have to take my drugs. What advice do you give?
- We hear that there is support that is given to HIV patients but we don't see this support. Where is this support delivered? How can we access this support?

**Reproductive goals & PMTCT & transmission risk**

- If I produce a child, do I need to share my ARVs [antiretrovirals] with that child or is the child given other drugs?
- We need some information on how to live with a discordant partner.
- How can a couple where a man is negative and a woman positive produce a kid?
- How can you help those women who are positive and receiving care but their husbands have refused to test?
- What can I do to produce a safe baby?

**Mental health**

- You talked about depression, how can we avoid it?
- I developed a mental problem after initiation of ARVs, which has caused me to isolate myself from the others and have food insecurity. How are you going to help?
- I have a child who is HIV positive and taking ARVs and always asks why he is taking the drugs, every time I keep on dodging him. How then shall I begin telling him that he has the virus? He is 9 years of age.

**Adherence**

- It was said that when someone misses drugs, the virus multiplies, when one starts his drugs, when does the virus go?

**General medical questions & HIV complications**

- When we start ARVs, we experience things like red lips, fatty abdomen, and sunken cheeks, and they persist. How can we avoid or manage them?
- Does alcohol affect the ARV drugs that we take?
- How come that the sexual urge increases when someone starts ARV drugs?
- Is it true that someone can be healed from HIV after being prayed for?

## Abbreviations

ART: antiretroviral therapy
IRC: institutional review committee
ISS: immune suppression syndrome
MUST: Mbarara University of Science and Technology
UARTO: Uganda Antiretroviral Rural Treatment Outcomes
UNCST: Uganda National Council of Science and Technology
